# Towards Human-like Walking with Biomechanical and Neuromuscular Control Features: Personalized Attachment Point Optimization Method of Cable-Driven Exoskeleton

**DOI:** 10.3389/fnagi.2024.1327397

**Published:** 2024-02-02

**Authors:** Yasheng Chen, Weiwei Yu, Abderraouf Benali, Donglai Lu, Siong Yuen Kok, Runxiao Wang

**Affiliations:** ^1^School of Mechanical Engineering, Northwestern Polytechnical University, Xi'an, China; ^2^LISV, Versailles Systems Engineering Laboratory, Université de Versailles Saint Quentin en Yvelines, Paris, France

**Keywords:** biomechanical analysis, rehabilitation for aging, cable-driven exoskeleton, neuroscience, cable attachment optimization, muscle force

## Abstract

The cable-driven exoskeleton can avoid joint misalignment, and is substantial alterations in the pattern of muscle synergy coordination, which arouse more attention in recent years to facilitate exercise for older adults and improve their overall quality of life. This study leverages principles from neuroscience and biomechanical analysis to select attachment points for cable-driven soft exoskeletons. By extracting key features of human movement, the objective is to develop a subject-specific design methodology that provides precise and personalized support in the attachment points optimization of cable-driven exoskeleton to achieve natural gait, energy efficiency, and muscle coordination controllable in the domain of human mobility and rehabilitation. To achieve this, the study first analyzes human walking experimental data and extracts biomechanical features. These features are then used to generate trajectories, allowing better natural movement under complete cable-driven exoskeleton control. Next, a genetic algorithm-based method is employed to minimize energy consumption and optimize the attachment points of the cable-driven system. This process identifies connections that are better suited for the human model, leading to improved efficiency and natural movement. By comparing the calculated elderly human model driven by exoskeleton with experimental subject in terms of joint angles, joint torques and muscle forces, the human model can successfully replicate subject movement and the cable output forces can mimic human muscle coordination. The optimized cable attachment points facilitate more natural and efficient collaboration between humans and the exoskeleton, making significant contributions to the field of assisting the elderly in rehabilitation.

## 1 Introduction

Due to advances in medical technology, improvements in living conditions, increased health awareness, and declining birth rates, the global population is experiencing rapid aging (Organization et al., 2020). As individuals age, they often encounter a gradual decrease in muscle mass and the quantity of muscle fibers, a condition referred to as muscle atrophy. Concurrently, the incidence of stroke, particularly among the elderly, rises significantly, with rates as high as 65% (Lo Coco et al., 2016). This muscle atrophy is typically attributed to various factors, including alterations in the nervous system, hormonal fluctuations, and reduced physical activity levels (Ziaaldini et al., 2017). These factors collectively contribute to the weakening of lower limb strength in older adults, thereby elevating the risk of falls, disability, and mortality (Thompson et al., 2021).

Existing research has demonstrated the high effectiveness of regular exercise in preventing and treating age-related skeletal muscle loss symptoms (Frizziero et al., 2016; Yoo et al., 2018; Shur et al., 2021). Furthermore, physical exercise shows promise in enhancing functional motor recovery by promoting neuroplasticity at various cellular and molecular levels (Pin-Barre and Laurin, 2015; Liang et al., 2021). To facilitate exercise for older adults, and improve their overall quality of life, robot technologies have found applications in the field of elderly care (Alnajjar et al., 2019), including the use of active exoskeleton robots (Li et al., 2021; Xiong et al., 2022) and assistive robots (Pino et al., 2015). To achieve the goal of “rehabilitation training in daily life” and meet the requirements for joint driving capability and movement precision, many internationally developed wearable rehabilitation exoskeletons rely on rigid components such as motors and gear reducers (Carnevale et al., 2018; Nakashima et al., 2020). However, these traditional rigid exoskeleton designs tend to be heavy, increasing the mass and inertia of the user's distal body parts and consequently elevating metabolic costs during movement. Notably, one significant drawback of these rigid exoskeleton designs is the issue of joint misalignment (Yeem et al., 2018). Additionally, wearing such exoskeletons often results in substantial alterations in the average pattern of muscle synergy coordination among participants (Li et al., 2019).

Inspired by human muscles and tendons, flexible drives systems such as pneumatics (Prasad et al., 2022), hydraulics (Glowinski et al., 2020), and cables (Prasad et al., 2022) offer distinct advantages in the design of exoskeletons, including high power density ratio, wide contraction range, rapid response, flexible structure, and safety. Among these options, lighter and simpler cable drives exhibit particular advantages in exoskeletons, as they efficiently replicate the biomimetic properties of human tendon-joints (Xiloyannis et al., 2021). An illustrative example is the cable-driven exoskeleton garment, known as an Exosuit, which employs electric motors to drive hip and ankle joint movements through Bowden cables, enabling flexible actuation for assisting in horizontal walking (De Rossi et al., 2022). Another innovative development is the HitExosuit, which features twisted cable actuators and artificial muscles based on steel cables, supplying essential force to each hip joint during stair climbing, providing assistance torque to the knee joints, which are most crucial during this activity (Zhao et al., 2019). In the context of stair ascent and descent, a soft exoskeleton system with an additional knee joint effectively reduces muscle activity, thus lightening the user's load (Lee et al., 2020). A wearable soft robotic exosuit incorporating a portable cable actuation system is introduced, applying hip abduction torque to potentially mitigate knee osteoarthritis progression by reducing external knee adduction moment (Yang et al., 2022). A portable waist-loaded soft exosuit, concentrated at the waist, effectively aids hip flexion during running, demonstrated through motion flexibility experiments (Chen et al., 2022). In Biao et al. (2023), a novel flexible Bowden cable-driven exoskeleton is introduced, with each joint controlled by a pair of antagonistic muscles. This design utilizes direct lower limb motion feedback to achieve a natural gait during walking.

However, biomechanical investigations have highlighted the importance of biarticular elements in enhancing energy efficiency during gravity-intensive daily activities, signifying the crucial role of biarticular muscles in the future of exoskeleton-driven systems (Sharbafi et al., 2018). An exemplary flexible wearable device in this context is the exoskeleton robot Myosuit, which employs biarticular and coordinated control methods, making it suitable for gravity-intensive daily activities like sitting transfers (Schmidt et al., 2017; Ganesan and Gupta, 2021). BATEX, a new exosuit, is inspired by human musculoskeletal systems and neural control, to enhance metabolic efficiency, adaptability, and overall effectiveness (Firouzi et al., 2021). Additionally, exosuits utilize flexible drive units with Bowden cables to connect the ankle joint and thigh, delivering active assistance during ankle plantar flexion, consequently reducing metabolic consumption and muscle output (He et al., 2023). These exosuits contribute significantly to assisting the elderly in various tasks across different scenarios. Nevertheless, it is worth noting that the attachment points or routing positions of most exoskeleton suits are typically determined based on experiential and intuitive knowledge, with only a limited number of exosuits being optimized for factors such as stiffness (Wu et al., 2019), cable tension (Grosu et al., 2018), joint torque (Prasad et al., 2023), or metabolic factors (Bardi et al., 2022). In Prasad et al. (2022) a promising design option is proposed for a lower limb rehabilitation exoskeleton. Its identification is based on tracking performance, model requirements, and forces exerted on the limb by its components.

On the other hand, the biomechanical characteristics of the human body encompass not only the interaction of biarticular muscles but also the coordination between monoarticular and biarticular muscles (Junius et al., 2017). Moreover, aging constitutes a systemic reorganization of the neuro-musculo-skeletal system, extending beyond the mere degeneration of muscles (Sleimen-Malkoun et al., 2014). As individuals age, changes in age-related neural circuits can lead to alterations in muscle coordination, prompting compensatory changes to adapt to new conditions (Guo et al., 2022). Fortunately, the aging brain retains the capacity to reorganize activation patterns between neural circuits, accommodating anatomical and physiological changes (Vernooij et al., 2016). As motor tasks become more complex, this expanded activation is typically more pronounced, resulting in the emergence of new muscle synergy patterns, which are closely associated with changes in white matter and neural plasticity within the brain (Singh et al., 2018). Therefore, rehabilitation strategies founded on muscle synergistic interactions hold great promise in inducing genuine recovery and enhancing neuro-muscular coordination (Hong et al., 2021).

In light of their ability to mimic human muscle coordination and facilitate its achievement, exoskeletons hold immense significance in assisting older adults in their rehabilitation journey. Additionally, the selection of attachment points for soft exoskeletons significantly impacts their comfort, energy efficiency, and wearability (Asbeck et al., 2014; Tonazzini et al., 2018). The activation patterns of lower limb muscles exhibit variations across different walking speeds (Arnold et al., 2013). The cable arrangement and attachment points should also be finely tuned to meet the demands of various Activities of Daily Living (ADL) (Joshi et al., 2022). Exosuit-assisted strategies that rely on individual measurements of muscle dynamics in specific tasks, coupled with personalized approaches for achieving natural and complete gait restoration, along with targeted feedback, enhance exosuit performance. Furthermore, these strategies promote the establishment of new neural control centers and stimulate the activation of the entire lower limb muscle group, thereby providing valuable feedback to the cerebral cortex (Biao et al., 2023).

To address the aforementioned requirements effectively, before the implementation of cable-driven exoskeleton system, three key problems need to be tackled:

① The method to extract the biomechanical and neuro-control features from human motion experimental data, providing a more natural and effective motion reference for the cable-driven exoskeleton.
② The cable-driven exoskeleton modeling method considering the dynamics of the elderly human model and cable-driven system as well.
③ The optimization method of cable attachment points to make the cable-driven exoskeleton realize human-like walking motion with biomechanical features (e.g., ZMP, COM, joint angles and torques trajectories) and neuromuscular control features (e.g., muscle output forces and muscle synergy patterns).

With these challenges in mind, this article proposes a subject-specific method for determining the attachment points of a cable-driven exoskeleton. This approach allows the generated forces of the cables to adapt to the biological synergy during motion, thereby facilitating neuroplasticity in patients with neurological impairments and facilitating the restoration of normal walking ability. Additionally, this method holds promise for the development of humanoid musculoskeletal assistive robots, as older adults tend to accept humanoid robots more readily (Choudhury et al., 2018; Feingold Polak et al., 2018). Musculoskeletal robots can assess the muscle strength of older adults during movement and obtain precise data throughout the rehabilitation process, enabling adjustments to rehabilitation plans based on individual circumstances and needs (Farhat et al., 2022).

## 2 Materials and methods

**Figure 1:**
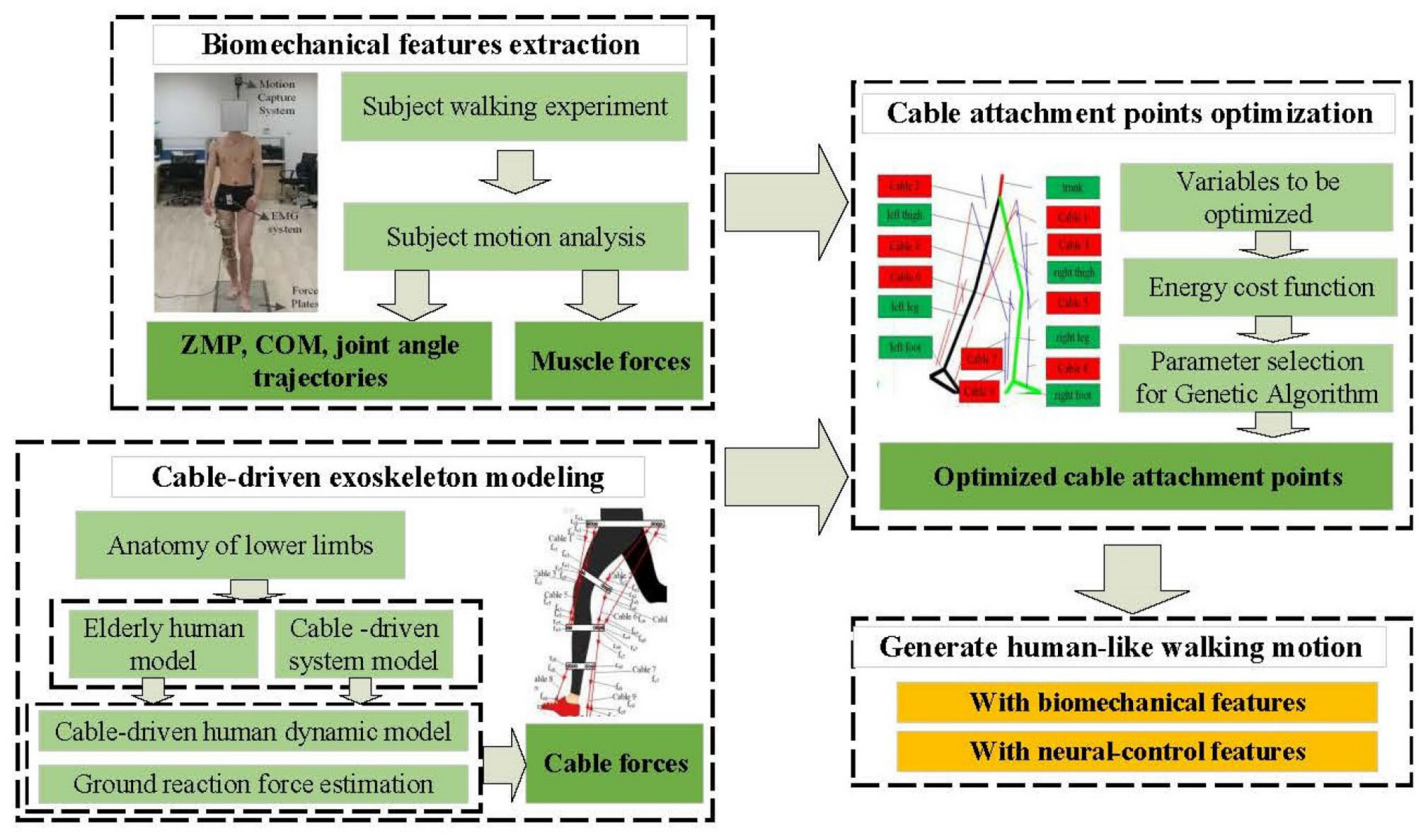
Subject-specific method for attachment points optimization of cable-driven exoskeleton.

With the continuous advancement of science and technology, exoskeleton robotic systems have made significant breakthroughs in improving the quality of life and providing assistance. This research aims to further drive the design of subject-specific cable-driven exoskeleton before its mechanical implementation to provide more human-like nature motion to support the fields of elderly human mobility and rehabilitation. The flowchart of the proposed approach is shown in Figure 1.

Firstly, subject walking experiments are performed, and the experimental data is analyzed to extract biomechanical features. These features are applied to generate trajectories for the elderly human model, aiming to achieve similar movements under complete cable-driven exoskeleton control. Additionally, the subject's neural-control features such as muscle forces and muscle activation order are calculated using electromyography (EMG) and dynamic data.

Next, based on neuroscience, an analysis of the roles and contributions of lower limb muscles during movement is conducted. Using elderly subject data, a human model is established, and the cable-driven system is determined. Separately, the lower limb biomechanics model for the elderly human model, the dynamics model for the cable-driven system, and the ground reaction force model are constructed to derive the dynamic model of cable-driven exoskeleton. The calculation of cable forces are calculated based on the specific application scenarios in which the human motion are totally activated by the cable-driven system.

Finally, the genetic algorithm (GA) is employed to optimize the attachment points of the cable-driven system, seeking attachment points that are well-suited for the human model, in order to make the system generate human-like walking motion with biomechanical features and neural-control features as well. In the following sections, the detailed materials and methods used in our research are delved into.

### 2.1 Modeling

Modeling forms the foundation of cable-driven exoskeleton optimization. In the following section, we will provide an in-depth examination of the modeling techniques and methods employed in our research.

#### 2.1.1 Biomechanical and neuromuscular control analysis of human lower limbs' motion

The lower limbs serve as the foundational support structure of the human body, maintaining an upright posture and facilitating movement. These lower limbs consist of three major joints: the hip joint, knee joint, and ankle joint. However, designing a lower limb exoskeleton that strictly adheres to the 14 degrees of freedom found in the human lower limb system is not only challenging but also demands extensive computational resources. According to Park's (2008) research, movements of the human upper limbs, including the arms, have minimal impact on sagittal dynamics. As a result, the human leg is simplified to incorporate 6 degrees of freedom in the sagittal plane, while the upper limbs, trunk, and head are collectively simplified as a single entity.

Human motion involves a complex interplay of more than 40 muscles that are relevant to various movements. However, attempting to incorporate all of these muscles into an exoskeleton system is not a practical endeavor. As previously mentioned, human joint movements are achieved through the complementary functions of monoarticular and biarticular muscles. Monoarticular muscles primarily generate unidirectional forces in the form of tension and are capable of performing pulling actions. To enable bidirectional motion, two monoarticular muscles are typically arranged as flexors and extensors. On the other hand, biarticular muscles, which span two joints, play a crucial role in human walking. They serve several vital functions, including the reduction of limb inertia during fast movements. They also contribute to gait stability, extension of the working range of motion, and optimization of the working point of monoarticular muscles. Additionally, biarticular muscles assist in maintaining limb balance, contribute to the control of leg swings, and have a positive impact on the development of running exercises. The consideration of neuroscience in this context adds to our understanding of the intricate neuromuscular control involved in human motion.

The results of neuroscience research indicate that in the sagittal plane, the knee and hip joints are driven by seven major muscles, which include four monoarticular muscles: iliacus (ILPSO) for hip flexion, gluteus maximus (GMAX) for hip extension, biceps femoris (BFS) for knee flexion, and vastus (VAS) for knee extension. There are also three biarticular muscles involved: rectus femoris (RF) for hip extension and knee flexion, hamstring (HAMS) for hip extension and knee flexion, and gastrocnemius (GAS) for knee flexion and ankle extension, as documented in the research by Hwang et al. (2018). Furthermore, two monoarticular muscles that drive the ankle joint have been selected for inclusion: the soleus (SOL) for ankle plantar flexion and the tibialis anterior (TA) for ankle dorsiflexion. These muscles play critical roles in controlling foot movements and maintaining balance during human locomotion, as outlined in the study by Di Giulio et al. (2009). These nine major muscles as the mimicry targets for the cable-driven exoskeleton are selected to drive the human model.

#### 2.1.2 Lower limb modeling of elderly: an example based on average data

As a result of the aging process, the bodies of elderly individuals undergo regressive changes, leading to noticeable alterations in their body size, particularly a decrease in height compared to their younger years. These aging-related changes also affect various other body dimensions. Consequently, the height dimensions of elderly individuals cannot be simply equated to those of the local average young population. For this study, we have utilized an example of average dimensions of male elderly individuals, which have been sourced from the research conducted in Hu et al. (2007). These dimensions include an average height of 1,657 mm, a weight of 67 kg, a thigh length of 448 mm, a calf length of 401 mm, an ankle height of 66 mm, a foot length of 241 mm, and a foot width of. These measurements provide a more accurate representation of the physical characteristics of elderly individuals. To further enhance the accuracy of our analysis, the estimation of the center of mass and moments of inertia for each body segment is based on Cheng et al. (2000). For a comprehensive breakdown of these parameters, please refer to Table 1.

**Table 1:** Parameters of human body model for elderly people.

**Body part**	**Length (mm)**	**Mass (kg)**	***I*_*x*_(*kg*·*m*^2^)**	***I*_*y*_(*kg*·*m*^2^)**	***I*_*z*_(*kg*·*m*^2^)**
Truck	Length: 742	40.2	1.42	0.25	1.29
Hips	Hip circumference: 963, hip breadth: 346	/	/	/	/
Thigh	Length: 448, thigh circumference: 521	9.11	0.16	0.020	0.16
Calf	Length: 401, width: 92	2.95	0.036	0.0043	0.035
Foot	Length: 241, width: 93, foot height: 66	1.34	0.012	0.018	0.016

Once the initial human body model has been established, the next step involves performing a kinematic analysis of the human body. In this analysis, the human model is regarded as a free-system configuration, and a set of generalized coordinates is defined as shown in Equation (1):


(1) $$\begin{array}{l} \text{q}={\left[x,y,q_{1},q_{2},q_{3},q_{4},q_{5},q_{6},q_{7}\right]}^{T} \end{array}$$

**Figure 2:**
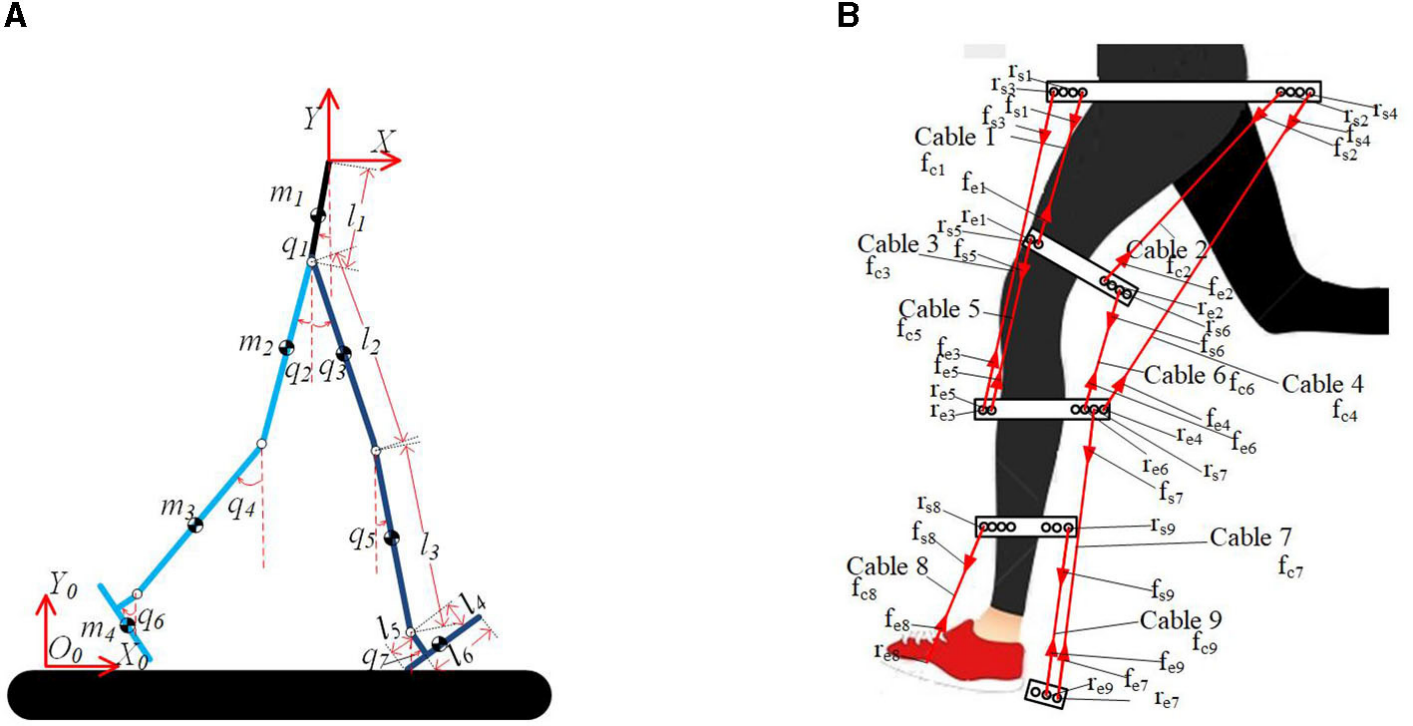
Human model and simplified cable-driven exoskeleton model. **(A)** Coordination of human model. **(B)** Simplified model of cable-driven exoskeleton.

In this set of coordinates, *x*,*y*,*q*_1_~*q*_7_ represent generalized coordinates as shown in Figure 2A. Additionally, there are parameters denoted as *l*_1_, *l*_2_, *l*_3_, *l*_4_, *l*_5_ that correspond to specific lengths in the human model: the length of the trunk, the length of the thigh, the length of the shank, the height from the foot to the ankle joint, the length of the heel, and the length of the toe. These parameters are illustrated in Figure 2A. The DH parameters are further detailed in Table 2. With these DH parameters and the known joint angles, it becomes feasible to calculate the kinematics of the human model, including the position, velocity, and acceleration of each component of the human body. Subsequently, the dynamic model will be analyzed in further detail, building upon the obtained kinematic information.

**Table 2:** D-H parameters in left leg.

**i**	**α_*i*−1_**	***a*_*i*−1_**	***d*_*i*_**	**θ_*i*_**
1	0	0	0	*q*_1_−π/2
2	0	*l*_1_	*l*_*w*_/2	*q*_2_−*q*_1_
3	0	*l*_2_	0	*q*_4_−*q*_2_
4	0	*l*_3_	0	*q*_6_−*q*_4_

#### 2.1.3 Cable-driven system modeling

According to the previous analysis, it is understood that nine major muscles of human leg play major role in the motion of sagittal plane. Therefore, in this chapter, nine cables have been set up, each designed to mimic the functions of these muscles, in order to drive the elderly human model to move like a healthy individual. The cable distribution is depicted in Figure 2B. The tension of the *i*_*th*_ cable is denoted as *f*_*ci*_, which is a scalar value. We set the attachment point closer to the trunk as the starting point, and the other as the ending point. The starting and ending points of the *i*_*th*_ cable on the human model are set as *r*_*si*_ and *r*_*ei*_. The forces applied at *r*_*si*_ and *r*_*ei*_ are represented as ***f***_*si*_, ***f***_*ei*_. The cables are configured as a massless rigid cable model with infinite stiffness. The properties to be defined for this model include a set of attachment locations and the minimum and maximum force bounds for the cables (Eden et al., 2018).

Consistent with the prior analysis, the resolution of cable tensions is profoundly affected by the selection of attachment points. Prior to identifying the optimal attachment points, we initially established the initial positions of cable attachment points based on the attachment points of lower limb muscles using a linear mapping. The initial attachment points are determined by linearly mapping human muscle attachment points to the attachment points in the human model. Muscle origin and insertion data are collected from eight males with an average height of 175 (±8.36) cm and an average age of 38.75 (±10.61) years (Shan, 2003). The distance between the hip joint and the knee joint averages 40.82 (±2.35) cm, while the distance between the knee joint and the ankle joint averages 38.00 (±2.37) cm.

Due to the fact that cables can only provide tension, the minimum force is set to 0. The maximum force values are determined by the driver. In this paper, we plan to use MYO-muscle to mimic the functions of muscles through the cables (Marques et al., 2013; Eden et al., 2018). Other structures have relatively small masses compared to the human model, and since this paper primarily focuses on cable forces and attachment point optimization, the modeling of other mechanical structures in the exoskeleton is intentionally disregarded. To calculate the cable forces in the exoskeleton, the cable dynamic model will be discussed in detail in the next section.

#### 2.1.4 Dynamic model

The cable-driven exoskeleton including human model is a system with multiple degrees of freedom, and it is crucial to establish its dynamic model and conduct relevant analyses. The Lagrange equation is employed to establish the system's dynamics equations:


(2) $$\begin{array}{l} F_{i}=\frac{d}{dt}\frac{\partial L}{\dot{\partial q_{i}}}-\frac{\partial L}{\partial q_{i}}\left(i=1,2,3...n\right) \end{array}$$

where *q*_*i*_ represents the generalized coordinates of joint *i* shown in Figure 2A, $\dot{q_{i}}$ is the corresponding velocity, *F*_*i*_ is the nonconservative force acting on the joint *i* of human model, and *n* is the number of links of human model. The Lagrangian function *L* is defined as the difference between the kinetic energy *K* and the potential energy *V* of the cable-driven exoskeleton including the human model: *L* = *K*−*V*.

The center of mass of the *i*_*th*_ link of human model in the generalized coordinate system is represented as $p_{i}={\left[x_{i}\left(q\right),y_{i}\left(q\right)\right]}^{T}$, which are obtained through direct kinematics. The velocity and angular velocity of the *i*_*th*_ link in the generalized coordinate system are represented as: $\dot{p_{i}}=J_{v_{i}}\dot{q}$ and $\omega_{i}=J_{\omega_{i}}\dot{q}$, *J*_*v*_*i*__ and *J*_ω_*i*__ are, respectively, the velocity Jacobian matrix and angular velocity Jacobian matrix. So the kinetic enery and potential energy can be calculated as $K=\overset{n}{\underset{i=1}{\sum }}\left(\frac{1}{2}{\dot{p_{i}}}^{T}m_{i}\dot{p_{i}}+\frac{1}{2}{\omega_{i}}^{T}R_{i}I_{i}R_{i}^{T}\omega_{i}\right)=\frac{1}{2}\dot{q^{T}}\sum_{i=1}^{n}\left(m_{i}J_{v_{i}}^{T}J_{v_{i}}+J_{\omega_{i}}^{T}R_{i}I_{i}R_{i}^{T}J_{\omega_{i}}\right)\dot{q}$, the potential energy $V=\overset{n}{\underset{i=1}{\sum }}m_{i}gy_{i}$. *I*_*i*_ is moments of inertia and *R*_*i*_ is the attitude transformation matrix of the link coordinate system relative to the world coordinate system. Clearly, *K* is a function of ***q*** and $\dot{q}$, while *V* is a function of ***q***. Then the Equation (2) can be deduced as


(3) $$\begin{array}{l} F_{i}=\frac{d}{dt}\frac{\partial K}{\dot{\partial q_{i}}}-\frac{\partial K}{\partial q_{i}}+\frac{\partial V}{\partial q_{i}}\left(i=1,2,3...n\right) \end{array}$$

The first term in the Equation (3) can be expanded as


(4) $$\begin{array}{l} \frac{d}{dt}\frac{\partial K}{\dot{\partial q_{i}}}=\frac{\partial }{\partial \dot{q_{i}}}\left(\frac{\partial K}{\partial \dot{q_{i}}}\right)\ddot{q_{i}}+\frac{\partial }{\partial \dot{q_{i}}}\left(\frac{\partial K}{\partial q_{i}}\right)\dot{q_{i}} \end{array}$$

We set $D=\overset{n}{\underset{i=1}{\sum }}\left(m_{i}J_{v_{i}}^{T}J_{v_{i}}+J_{w_{i}}R_{i}I_{i}R_{i}^{T}J_{w_{i}}\right)$, the Equation (4) can be further written as shown in Equation (5)


(5) $$\frac{d}{dt}\frac{\partial K}{\dot{\partial q_{i}}}=\sum_{j=1}^{n}D_{ij}{\ddot{q}}_{i}+\sum_{j,k=1}^{n}\frac{\partial D_{kj}}{\partial q_{i}}{\dot{q}}_{j}{\dot{q}}_{k}$$

The second term in the Equation (3) can be expanded as shown in Equation (6)


(6) $$\frac{\partial K}{\partial q_{i}}=\frac{1}{2}\sum_{j,k=1}^{n}\frac{\partial D_{kj}}{\partial q_{i}}{\dot{q}}_{j}{\dot{q}}_{k}$$

Set $\Gamma_{ijk}=\frac{1}{2}\left(\frac{\partial D_{ij}}{\partial k}+\frac{\partial D_{ik}}{\partial j}+\frac{\partial D_{kj}}{\partial i}\right)$

The analytical expression of the dynamic equation is shown in Equation (7)


(7) $$\begin{array}{l} D\left(q\right)\ddot{q}+C\left(q,\dot{q}\right)\dot{q}+G\left(q\right)=F \end{array}$$

*D* represents the mass-inertia matrix, while $C_{i,j}=\overset{n}{\underset{k=1}{\sum }}\Gamma_{ijk}\dot{q_{k}}$ and $G=\frac{\partial V}{\partial q}$ represent the centrifugal-coriolis and gravitational terms, respectively. The nonconservative force *F* can be expressed as shown in Equation (8):


(8) $$F=\tau +J_{grf}^{T}\cdot f_{grf}+J_{\text{c}}^{T}\cdot f_{c}$$

**τ** represents the human joint drive torques. In our application case of the exoskeleton, we suppose that elder patient's lower limb movement is entirely driven by cable-driven exoskeleton, the patient joint drive torques **τ** can be set to zero. ***f***_*grf*_ represents the ground reaction force, ***J***_*grf*_ is the Jacobian matrix that relates the ground reaction force to the joints. ***f_c_*** denotes the cable force vector, which should be positive. Additionally, *J*_*c*_ is the Jacobian matrix that establishes the relationship between the cable force vector and the joints. ***f***_*grf*_, *J*_*grf*_, ***f***_*c*_, and *J*_*c*_ will be elaborated in detail in the following sections.

##### 2.1.4.1 Dynamic model of cable-driven system

To calculate the Jacobian matrix, *J*_*c*_, the following steps are taken. As shown in Figure 2B, *r*_*si*_ represents the attachment starting point of the *i*_*th*_ cable, and ***f***_*si*_ represents the tension of the *i*_*th*_ cable acting on the attachment starting point. Similarly, *r*_*ei*_ represents the attachment ending point of the *i*_*th*_ cable, and ***f***_*ei*_ represents the tension of the *i*_*th*_ cable acting on the attachment ending point. The unit vectors of the *i*_*th*_ cable's attachment points are defined by *r*_*si*_ and *r*_*ei*_. Additionally, the cable tension is represented as *f*_*ci*_, which is a scalar. The forces acting on *r*_*si*_ and *r*_*ei*_ can be expressed as $f_{si}=f_{ci}\cdot \hat{I_{i}}$ and $f_{ei}=-f_{ci}\cdot \hat{I_{i}}$, respectively, where $\hat{I_{i}}$ is a unit direction vector as shown in Equation (9).


(9) $$\begin{array}{l} {\hat{\text{I}}}_{i}=\frac{r_{\text{ei}}-r_{si}}{\sqrt{{\left(r_{ei}-r_{si}\right)}^{T}\left(r_{ei}-r_{si}\right)}} \end{array}$$

Component force *F*_*ij*_ of ***f***_*si*_ and ***f***_*ei*_ acting on *j*_*th*_ joint are expressed as shown in Equation (10):


(10) $$F_{ij}={\left(\frac{\partial r_{si}}{\partial q_{j}}\right)}^{T}f_{si}+{\left(\frac{\partial r_{ei}}{\partial q_{j}}\right)}^{T}f_{ei}={\left(\frac{\partial r_{si}}{\partial q_{j}}-\frac{\partial r_{ei}}{\partial q_{j}}\right)}^{T}\cdot {\hat{\text{I}}}_{i}\cdot f_{ci}$$

So the Jacobin matrix ${J_{c}}^{T}$ is expressed as shown in Equation (11)


(11) $$\begin{array}{l} J_{c}^{T}=\left[\begin{array}{ccc} {\left(\frac{\partial r_{e1}-\partial r_{s1}}{\partial q_{1}}\right)}^{T}\cdot {\hat{\text{I}}}_{1} & \cdots & {\left(\frac{\partial r_{ei}-\partial r_{si}}{\partial q_{1}}\right)}^{T}\cdot {\hat{\text{I}}}_{i}\\ \vdots \\ {\left(\frac{\partial r_{e1}-\partial r_{s1}}{\partial q_{j}}\right)}^{T}\cdot {\hat{\text{I}}}_{1} & \cdots & {\left(\frac{\partial r_{ei}-\partial r_{si}}{\partial q_{j}}\right)}^{T}\cdot {\hat{\text{I}}}_{i} \end{array}\right] \end{array}$$

##### 2.1.4.2 Estimation of ground reaction force

The ground reaction force on the standing leg during the single supporting phase is calculated based on both inertial force and gravity as shown in Equation (12),


(12) $$\{\begin{array}{ll} F_{grf} & =\sum \left(m_{i}{\ddot{p}}_{i}-m_{i}g\right)\\ M_{grf} & =\sum (\frac{d}{dt}(I\omega )-(m_{i}{\ddot{p}}_{i}-m_{i}g)\times r_{i}) \end{array}$$

**Figure 3:**
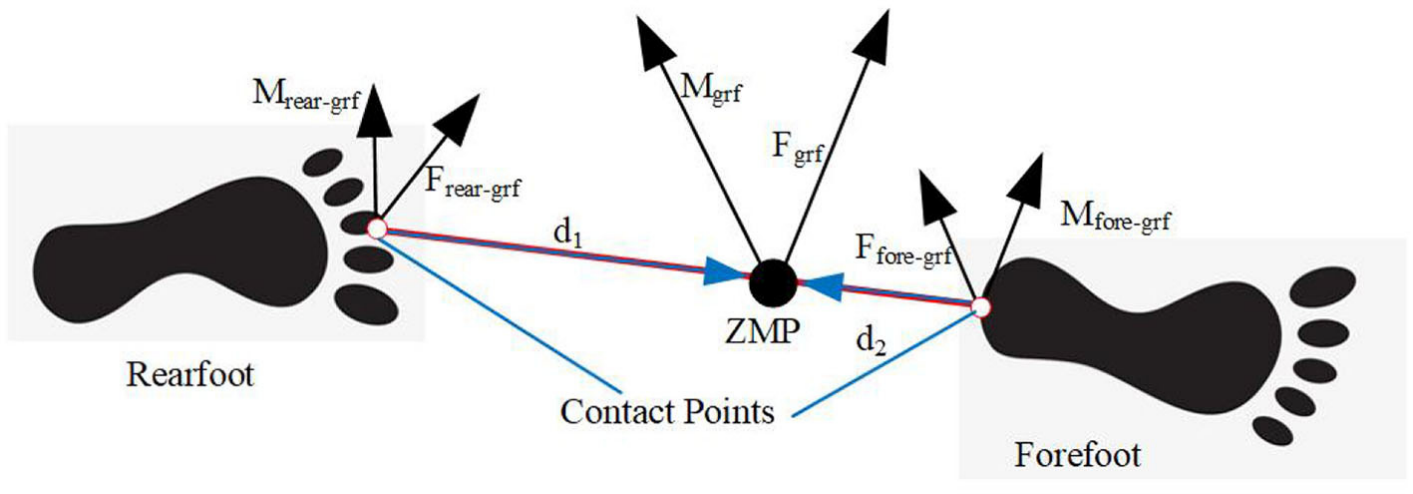
Partition of ground reaction forces.

In the double-supporting phase, the ground reaction forces are distributed between both legs. The distribution of this load is approximated using a weighted linear relationship between the two contact points on the foot and the position of the Zero Moment Point (ZMP) (Xiang et al., 2009). As shown in Figure 3, ***F***_*fore*−*grf*_, ***M***_*fore*−*grf*_ and ***F***_*rear*−*grf*_, ***M***_*rear*−*grf*_ represent the ground reaction forces on the forefoot and rearfoot, respectively. ***d***_1_ and ***d***_2_ represent the vector from the forefoot and rearfoot contact points to the ZMP, respectively. The trajectory of ZMP is determined based on human motion, which will be elaborated on in the following section.


(13) $$\begin{array}{l} F_{fore-grf}=\frac{\left||d_{2}|\right|}{\left||d_{1}||+||d_{2}|\right|}F_{grf}\\ M_{fore-grf}=\frac{\left||d_{2}|\right|}{\left||d_{1}||+||d_{2}|\right|}M_{grf}+d_{1}\times F_{fore-grf}\\ F_{rear-grf}=\frac{\left||d_{1}|\right|}{\left||d_{1}||+||d_{2}|\right|}F_{grf}\\ M_{rear-grf}=\frac{\left||d_{1}|\right|}{\left||d_{1}||+||d_{2}|\right|}M_{grf}+d_{2}\times F_{rear-grf} \end{array}$$

The interaction force between the human model and the ground can be obtained according to the force and moment on each leg of the human model in Equation (13) (Xiang et al., 2009);

During the single supporting phase, the contact point between the foot and the ground is modeled by Φ(***q***) as shown in Equation (14).


(14) $$\begin{array}{l} J_{grf}=\frac{\partial \Phi \left(q\right)}{\partial q} \end{array}$$

During the double supporting phase, the contact points between the foot and the ground are Φ_*fore*−*foot*_(***q***) and Φ_*rear*−*foot*_(***q***).

#### 2.1.5 Calculation of cable forces

The resulting dynamic model becomes a redundant dynamical system, leading to multiple solutions during the calculation of cable force. Consequently, the inverse dynamic problem is typically formulated as an optimization problem. Taking into account the constraints of positive cable force, the optimal set of cable forces can be determined by solving Equation (15).


(15) $$\begin{array}{l} f_{c}^{*}=\mathrm{argmin}Q(f_{c})\\ \text{ s.t. }D(q)\ddot{q}+C(q,\dot{q})\dot{q}+G(q)-J_{grf}^{T}f_{grf}=J_{\text{c}}^{T}\cdot f_{c}\\ f_{\text{min}}\leq f_{c}\leq f_{\text{max}} \end{array}$$

The minimum and maximum bounds on the cable force are represented by the vectors ***f***_*min*_ and ***f***_*max*_, respectively. A typical objective function $Q\left(f_{c}\right)=\overset{i}{\underset{m}{\sum }}f_{i}^{2}$ is selected to achieve a desired goal (Lau et al., 2016). The optimization is solved in interior point method. In next section, the human walking study is conducted in order to complete model analysis.

### 2.2 Human walking experiment and biomechanics features extraction

The daily activities require a wide range of lower limb movements, including walking, climbing stairs, bending, standing and sitting, walking, and changing clothes. Among these activities, balance-recovery stepping is a particularly common movement, and is considered a typical daily activity (Liu and Lockhart, 2009). Therefore, this chapter primarily focuses on generating motion data for the human model, specifically pertaining to the balance-recovery stepping motion observed in healthy individuals. Generating biomimetic gait is a complex proposition, involving not only kinematics but also dynamics and biomechanics. Many researchers have established a strong correlation between specific characteristics of human gait and aspects of walking stability and energy efficiency (Chen et al., 2017). Consequently, experiments on human walking was been designed to explore these characteristics through motion analysis. In addition, the muscle forces of the subject was also be analyzed to serve as an evaluation criterion for the performance of the human model driven by the exoskeleton.

#### 2.2.1 Human walking experiment

**Figure 4:**
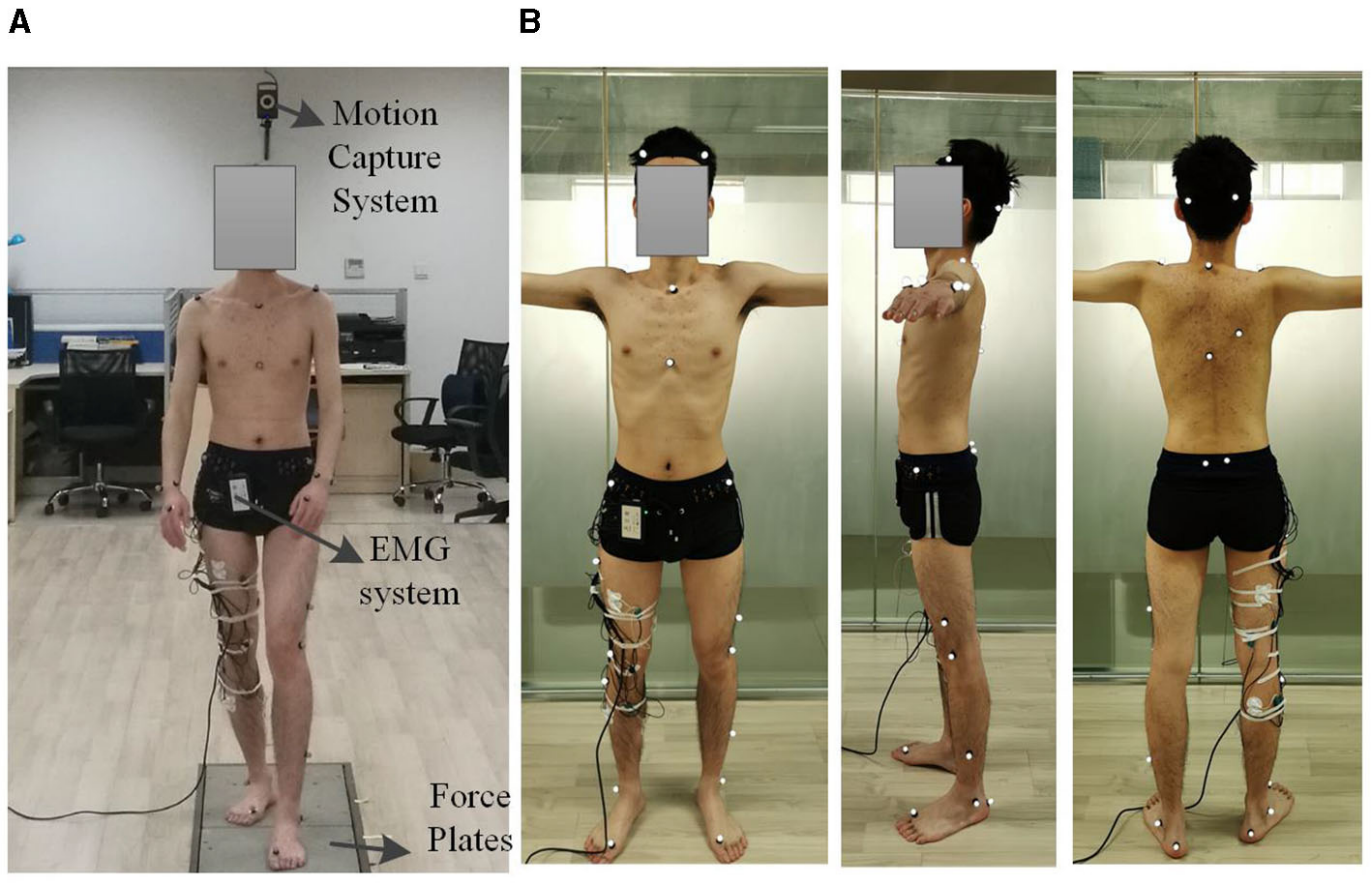
Experimental setup. **(A)** Motion capture system. **(B)** The position of the reflective markers.

The experimental environment is depicted in Figure 4A. It includes three force plates (40 × 60 cm, AMTI, USA) embedded in the floor to record ground reaction forces. Over 30 reflective skin markers (9-mm diameter) were strategically placed at joint locations and other significant anatomical landmarks. The selection of marker locations aligned with those in the Gait2354 Oensim model and includes markers at the acromion process of the scapula, anterior superior iliac spine, front thigh, lateral thigh, medial knee (medial femoral condyle), lateral knee (lateral femoral condyle), front shank, lateral shank, medial malleolus, lateral malleolus, heel (calcaneus), head of the fifth metatarsal, head of the first metatarsal, and tip of the first toe. These markers were placed on both the right and left sides of the body, as well as on the top of the head, top of the sacrum, and sternum shown in Figure 4B.

To collect kinematic data, a twelve-camera motion capture system (VICON T40S, Oxford, UK) was employed to capture data at a 100 Hz rate, while force-plate data was collected at 1,000 Hz. The analysis of the acquired experimental data was conducted using Opensim. In addition to the motion capture experiment, surface electromyography (EMG) data were also collected. As described in the previous section, six muscle signals were chosen for EMG recording, specifically the medial femoral muscle, rectus femoris, biceps femoris long head, gastrocnemius Lateralis, soleus muscle, and tibialis anterior muscle. The placement of electrodes follows the guidelines established in Stegeman and Hermens (2007). An eight-channel EMG acquisition box (Biovison, 8 Chanel, Germany), was utilized to capture EMG signals, with two of its channels synchronized with the VICON system at a sampling rate of 1,000 Hz.

To enhance the resemblance of the exoskeleton-driven human's motion to that of healthy individual, an experiment was conducted. The experiment involves a healthy adult male subject, with height of 1.78 m and weight of 56 kg, and without lower limb injury or illness. The subject was instructed to assume a double-stance position, with one foot placed in front of the other, each foot in contact with a force plate. The subject's arms hanged naturally at their sides. Upon receiving an “all set” signal, the participant initiated the gait and proceeded to walk straight ahead to the end of the walkway. The speed, height, and step length were determined under the most comfortable conditions.

The subject was fully informed about the experiment, and the experiment was being conducted following the review by the Institutional Ethics Review Board at Northwestern Polytechnical University (No. 202302009).

#### 2.2.2 Motion biomechanics analysis

After obtaining experimental data, the next step is to analyze the motion data to extract biomechanical features. The subject's joint angles are obtained through kinematic simulation using motion capture data from markers and plantar force data. Inverse dynamics simulation is then performed to calculate joint torques, and based on the plantar force data, the ZMP trajectory can be derived.

**Figure 5:**
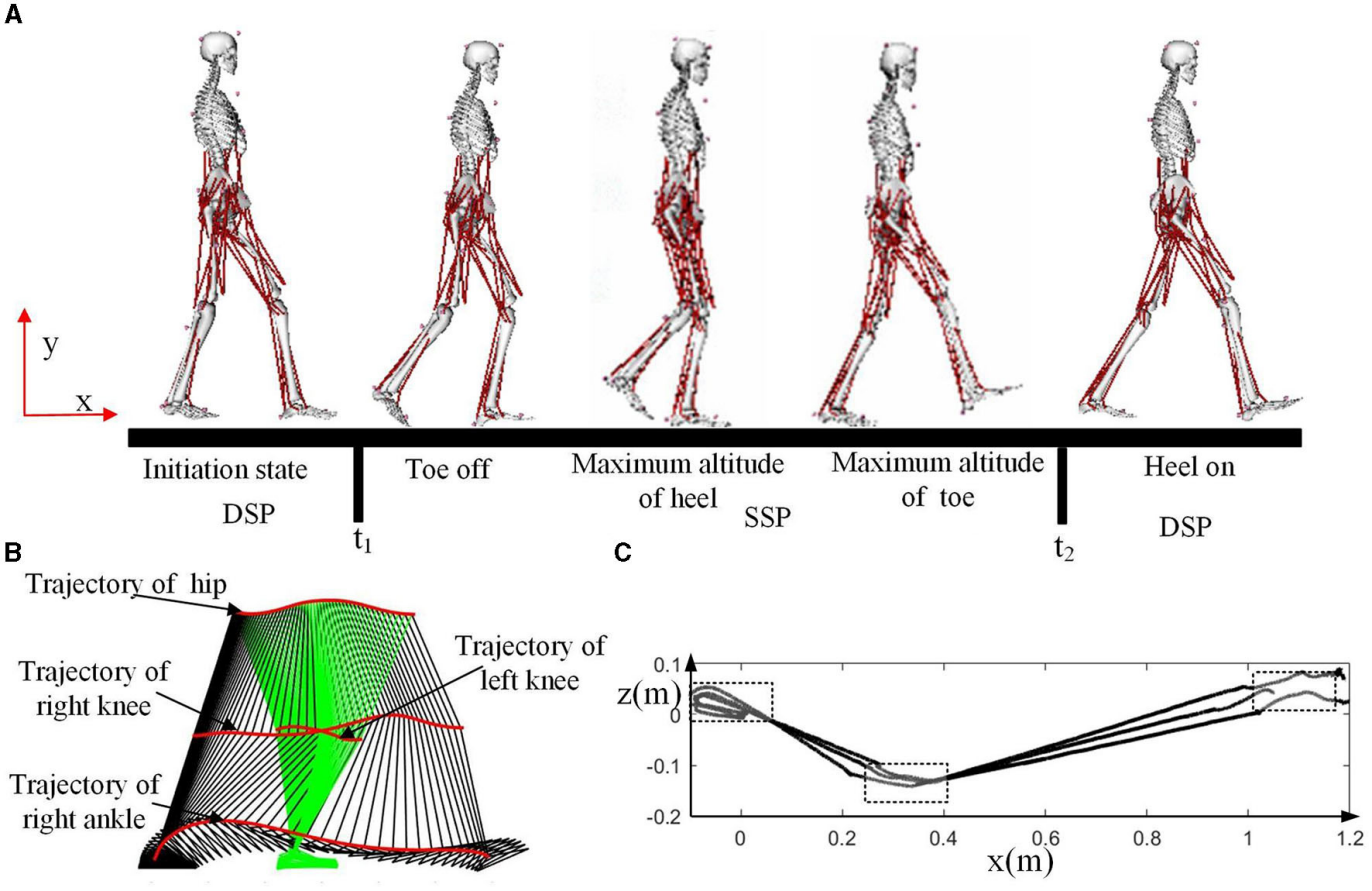
Motion analysis for the walking experiment. **(A)** Some key frames during the motion. **(B)** Snapshot of the motion. **(C)** ZMP trajectory of the three experiments.

Figure 5A illustrates some key frames during the motion, such as toe off, maximum altitude of heel, maximum altitude of toe, and heel on. At time t1, the toe-off event occurs, and the motion before t1 is in the double support phase (DSP). At time t2, the heel makes contact with the ground, and this phase is also considered as the double support phase (DSP). At the conclusion of the single support phase (SSP), the supporting foot pivots around its toe, while the swinging foot rotates around its heel upon contact with the ground. It's important to note that the heel of the swinging foot touches the ground before the heel of the supporting foot lifts off. The phase between t1 and t2 is the single support phase (SSP).

The snapshot of the motion is presented in Figure 5B, with green indicating the stance leg (left leg in this case) and black representing the swing leg (right leg in this case). There are four trajectories shown in the figure, with the ankle joint of the stance leg not displayed as its range of motion is limited. Evidently during walking, the hip joint undergoes a subtle, periodic up-and-down motion in the vertical direction. This periodic motion in the upper body leverages gravity to provide forward momentum for the body while reducing excessive energy consumption caused by knee joint bending. This results in an overall decrease in energy expenditure during walking (Zhu et al., 2016).

The ZMP trajectory is illustrated in Figure 5C, a vital indicator of human balance. During the initiation of forward walking, the ZMP initially moves backward, and the center of gravity shifts rearward to store energy. Subsequently, the torso swings forward, and the center of gravity shifts forward, initiating forward movement. During the single support phase, the Zero Moment Point (ZMP) safely progresses along the direction of the foot. In the double support phase, the ZMP moves in a straight line from the rear foot to the front foot. In the process of generating humanoid gait, it's essential to consider key features of human motion, such as the heel strike and toe-off of the feet, the movement of the center of gravity, and the trajectory of the ZMP.

#### 2.2.3 Human-like trajectory generation based on biomechanics features

This section is dedicated to the generation of motion trajectory for the human model driven by expskeleton. The process of generating humanoid trajectories is based on the methodology outlined in Boutin et al. (2011).

Initially, the joint angles obtained from OpenSim are directly mapped to the human model. However, this process can generate incorrect positions due to kinematic mismatches between the experimental subject and the elderly human model. Consequently, it becomes necessary to adjust the incorrect foot positions. The principles guiding this adjustment process are as follows: ensure that the human model's feet do not intersect with the ground and that the model does not slide during motion.

After the adjustment, key phases in the sequence are extracted, such as the moments when the heel strikes the ground and when the toe leaves the floor as shown in Figure 5A. The generated foot trajectories closely resemble those of the human body, ensuring that the angles of toe-off and heel strike are similar to those of the subject. The trajectories of the feet at these moments are linearly fitted to maintain the continuity of position, velocity, and acceleration. While these generated trajectories maintain a similar gait frequency to that of subject, the walking step length is adjusted based on the size of the human model.

To ensure model balance, it is crucial to control the ZMP trajectory. As previously analyzed, the ZMP remains inside the supporting leg and advances in the direction of the foot during the single stance phase. In the double stance phase, the ZMP moves in a straight line from the rearfoot to the forefoot. Based on this analysis and the previously generated feet trajectories, the ZMP trajectory of human model is planned. Although it is challenging to derive joint angles directly from ZMP trajectories, it is possible to calculate joint angles from the Center of Mass (CoM) using inverse kinematics simulation. Human motion analysis indicates that the CoM moves up and down during movement, and this information can be used in human-like motion generation for exoskeleton. An inverted pendulum model can be employed to find a CoM trajectory that ensures the ZMP follows the reference trajectory (Fayong et al., 2014).

After obtaining these values, optimization is performed using a damped least squares method to adjust the joint angles while taking into account the constraints related to the Center of Mass (CoM) and foot motion. This optimization process allows us to derive the desired trajectory for the human model to be driven by the cable-driven exoskeleton.

#### 2.2.4 Neuromuscular control analysis

In the realm of cable-driven exoskeleton robot system, a key focus lies in understanding and validating the neuromuscular control of human muscle forces during different activities. The human lower limb's muscular and skeletal system exhibits a high degree of redundancy, meaning that various muscle combinations and joint movement patterns can accomplish similar tasks. In such scenario, the use of Neuro-Cybernetics control methods can more effectively simulate and comprehend how the human biological nervous system coordinates muscles to perform motor tasks (Wong, 2023). To validate the biomechanics of these systems and understand muscle activation during human motion, the CEINMS toolbox is employed, as detailed in Pizzolato et al. (2015).

To achieve this, electromyogram (EMG) signals collected from the subject play a pivotal role. These signals are processed meticulously, following a structured approach. Initially, a zero-lag fourth-order recursive Butterworth high-pass filter is applied, efficiently eliminating noise from the raw EMG data. Subsequent steps involve full-wave rectification and low-pass filtering using a Butterworth filter with 6 Hz cutoff frequency, adhering to established techniques (Lloyd and Besier, 2003). These processed EMG signals are then mapped onto Musculotendon Units (MTUs), effectively associating specific muscle groups with their corresponding activation levels. This mapping is a vital bridge connecting EMG data to the intricacies of muscle dynamics. Further insights into the biomechanics are gleaned by calculating joint angles, Musculotendon Unit (MTU) lengths, and moment arms. Inverse kinematics aids in determining joint angles, while inverse dynamics calculations provide the necessary MTU parameters. OpenSim's muscle analysis tools prove instrumental in these computations. Crucially, the human body exhibits variations in muscle parameters across individuals, necessitating a meticulous calibration process. This step aims to optimize parameters within the Neuromusculoskeletal (NMS) model. Employing a calibration loop, the process minimizes discrepancies between estimated joint moments, derived from the NMS model, and experimental joint moments, directly measured from the subject. To initiate this calibration, initial parameters for the MTUs are drawn from a standardized model, such as the Gait2354 model within OpenSim. With successfully calibrated parameters, the estimation of Musculotendon Unit (MTU) forces becomes possible. This estimation considers calibrated MTUs, muscle excitations (activation levels), MTU kinematics (lengths and moment arms), and external joint moments. The culmination of these intricate steps provides valuable insights into muscle activation and forces during various human activities.

These insights, founded on rigorous calibration and meticulous analysis of EMG signals, serve as pivotal neuromuscular control validation data for cable-driven exoskeleton robot systems, enriching our understanding of human locomotion and facilitating the development of advanced robotic systems.

### 2.3 Optimization method of cable attachment points of exoskeleton

In the previous section, the cable attachment points of the cable-driven system were directly mapped from the human muscle attachment points data and need to be adjusted to better suit our requirements. To optimize these attachment points for improved cable performance that closely simulates the muscles of a healthy individual, a classic intelligent optimization algorithm, the genetic algorithm, was employed to determine the most suitable cable attachment points under conditions resembling human motion.

Within the genetic algorithm, the design variables pertain to the coordinates of the cable attachment points. As the muscle origin and insertion points vary among individuals, the cable attachment points will also vary within a certain interval (Shan, 2003). The upper and lower limits of these design variables are determined by appropriately expanding this interval. To enhance performance, it is crucial to define an appropriate evaluation function. For this purpose, we chose energy efficiency, which is a critical aspect of human dynamic walking and a significant indicator for exoskeleton systems (Roberts et al., 2017).

In this study, to calculate the energy consumption of Cable-driven exoskeleton System, we assume that cable tension is achieved through the use of MYO-muscles. The MYO-muscle is driven by a Maxon brushless motor with a power rating of 100 watts and is equipped with a Hall sensor. However, in the real system implementation, if different type of cable-driven artifical muscle is selected, the corresponding parameters in Equation (16) should be adjusted accordingly. The total energy consumption (*E*) is calculated as follows (Zhu et al., 2016):


$$\begin{array}{l} \;E=\int P_{\text{motor }}dt=\int u^{T}idt≅\int \sum_{i\in \{L,R\}}(C_{m}\tau_{i}^{T}{\dot{q}}_{i}+C_{t}\tau_{i}^{T}\tau_{i}\\ +C_{v}{\dot{q}}_{i}^{T}{\dot{q}}_{i})C_{m}=\frac{\left(2RF+BK\right)}{{\left(\gamma K\right)}^{2}},C_{t}=\frac{R}{{\left(n\gamma K\right)}^{2}},C_{v}=\frac{n^{2}(FR+BK)F}{K^{2}} \end{array}$$

*R*, *B*, *K*, *F*, γ, and *n* represent the armature resistance, back EMF constant, torque constant, viscous friction coefficient, gear efficiency, and gear ratio of the motor of the joint, respectively. These values can be obtained from the Maxon official website.

Taking the energy consumption as the optimization target, the Genetic Algorithm (GA) is selected to optimize the cable attachment points of exoskeleton system. Finally, the parameters for the Genetic Algorithm are configured as follows: Population size: 1,000, Number of terminating evolutionary generations: 100, Selection Probability: 0.7, Probability of crossover: 0.8, Probability of mutation: 0.08. These parameters define how the genetic algorithm operates during the optimization process to find the most suitable cable attachment points for the cable-driven exoskeleton system generating human-like motion.

## 3 Results and analysis

The exoskeleton dynamic simulation process including the elderly human lower limb modeling (Section 2.1.2), cable driven system modeling (Sections 2.1.3 Section 2.1.4), cable force calculations (Section 2.1.5), human-like walking trajectories generation (Section 2.2.3), and cable attachment points optimization (Section 2.3), were performed using MATLAB. The genetic algorithm utilized parallel computing to accelerate the calculations and took ~42,101 s to complete. The calculation of the subject's muscle forces was conducted with the CEINMS toolbox and took ~10 h.

**Figure 6:**
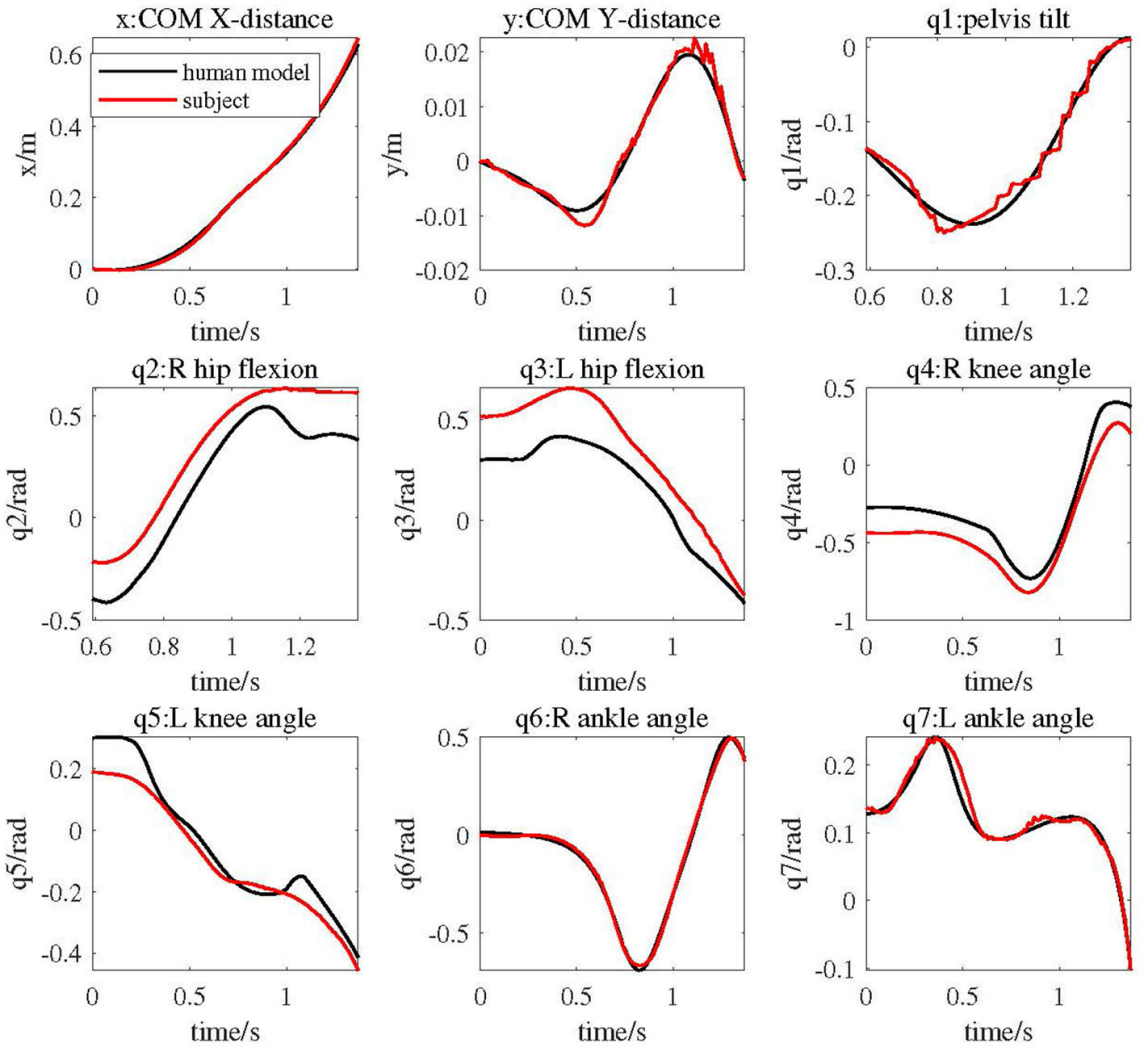
COM and joint trajectories of elderly human model driven by cable exoskeleton compared with experimental subject's data.

In Figure 6, the red lines represent the subject's COM trajectories and joint trajectories in the walking experiment, while the black lines depict the motion of the elderly human model driven by the cable exoskeleton, closely resembling human-like movements. From the dynamic simulation results, it's evident that the generated COM and joint trajectories of human model closely matches that of the experimental subject. The motion trends of the human model's torso closely follows the subject's trajectory but with smoother pattern. Notably, the entire trajectory of the human model's left leg aligns more closely with the subject's trajectory compared to the right leg.

**Figure 7:**
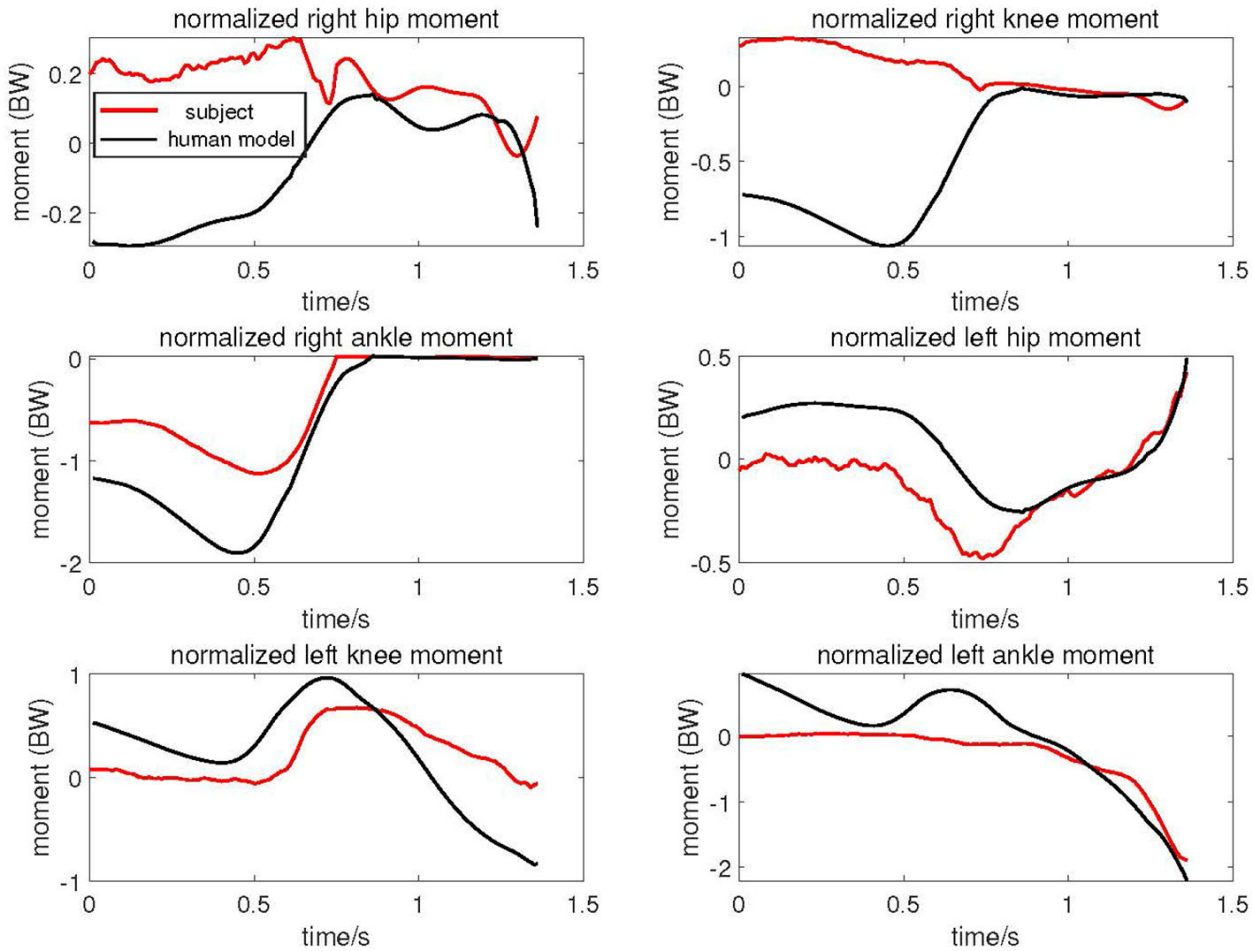
Joint torque of elderly human model driven by cable exoskeleton compared with experimental subject.

As shown in Figure 7, the red lines represent the subject's joint torques calculated by Opensim, while the black lines represent the joint angular torques of the human model driven by the exoskeleton during the same motion. All torques have been normalized based on body weight. The figure illustrates that the trends in joint torques for the human model and the subject are generally similar. Specifically, the joint torque trends at the right knee, left hip, left knee, and left ankle are very closely aligned. Prior to *t* < 0.75, the joint torques of the right leg are notably higher than those of the subject, whereas the joint torques of the left leg are lower. Subsequently, after *t* > 0.75s, the joint torque trends for both the left and right legs of the human model closely resemble the subject's joint torque changes.

**Figure 8:**
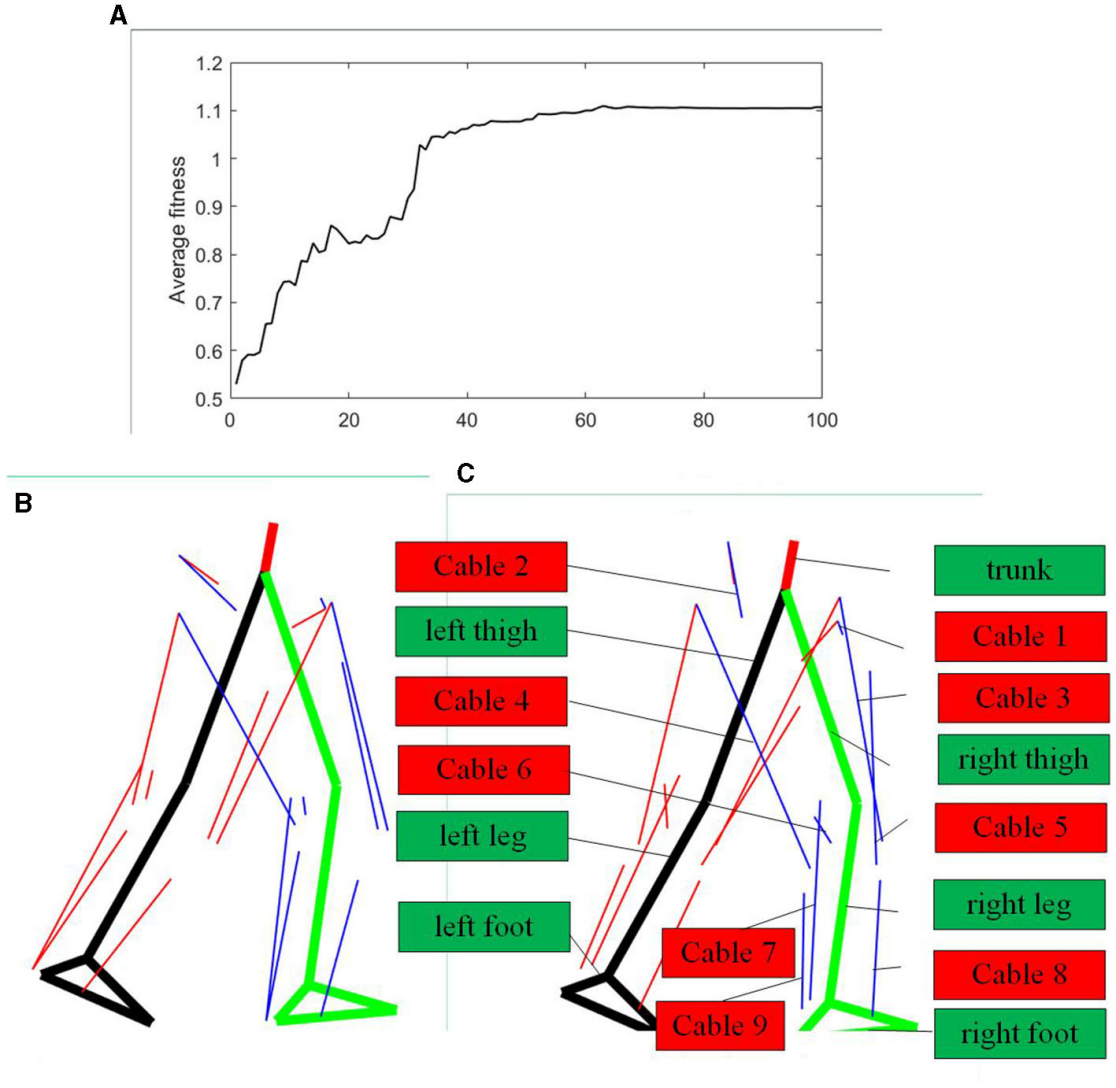
Cable attachment points optimization results. **(A)** Average fitness of the genetic algorithm. **(B)** Initial attachment points of cable-driven system. **(C)** Optimized attachment points of cable-driven system.

The average fitness of the genetic algorithm is presented in Figure 8A. It is noticeable that the average fitness converges at around the 60th generation. Figure 8B showcases the initial cable attachment point distribution, while Figure 8C presents the attachment point distribution after optimization. The blue outlines in Figure 8B represent various parts of the human model, including the trunk, thighs, lower legs, and feet, while the red outlines indicate that cable 1 to cable 9 are configured according to Figure 2B. The content in Figures 8B, C is identical, except for the specific cable attachment points' positions. In these figures, the thick black line corresponds to the left leg of elderly human model, the thick green line represents the right leg, the thin red lines symbolize the cables attached to the left leg, and the thin blue lines denote the cables attached to the right leg. It is evident that a significant difference exists between the initial attachment point distribution and the distribution after optimization. After optimization, the cable attachment points of the hip and knee joint extend to some degree in the direction along the lower limb, with minimal changes in the radial direction of the lower limb. Notably, the attachment points of the cable corresponding to the calf muscle after optimization are positioned closer to the ankle joint, whereas the attachment point of the cable corresponding to TA muscle moves farther away from the ankle joint.

**Figure 9:**
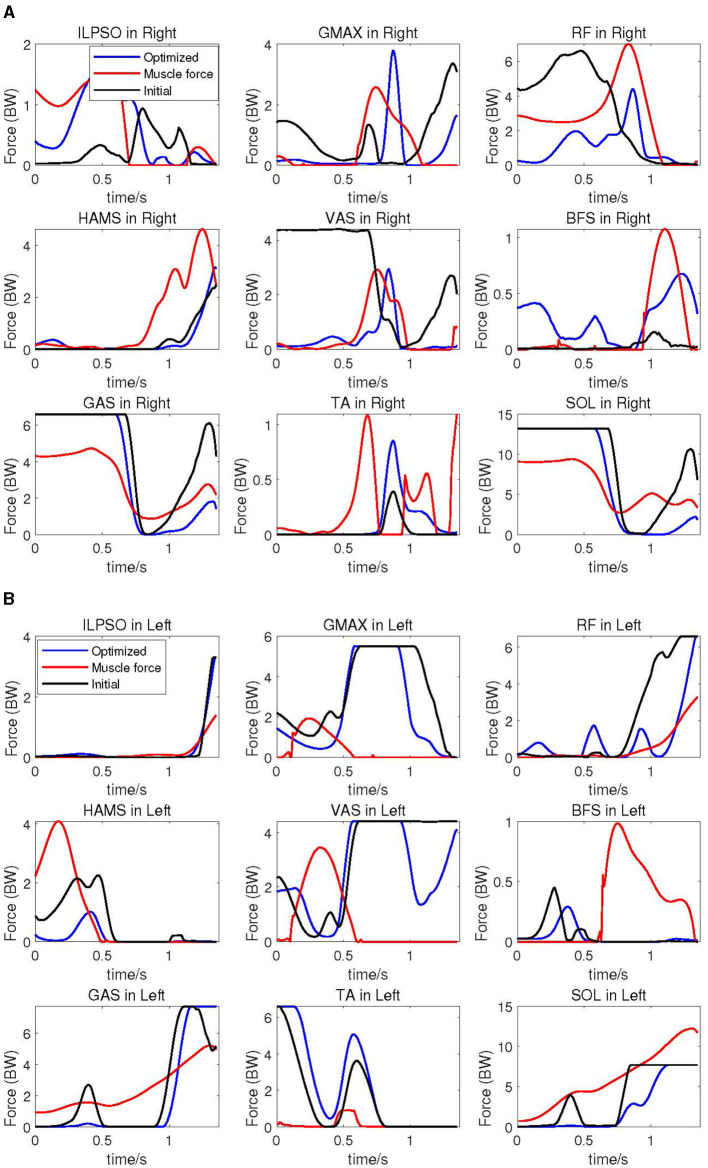
Corresponding cable forces before and after optimization, compared with experimental subject's muscle forces. **(A)** Corresponding cable forces of right leg compared with subject's muscle forces. **(B)** Corresponding cable forces of the left leg compared with subject's muscle forces.

In Figure 9A, the cable forces imitating specific muscle of right leg in various conditions are depicted. In this figure, the black line represents cable forces under the initial cable configuration, the blue line represents cable forces after optimization, and the red line represents the subject's muscle forces calculated using the CEIMS toolbox based on experimental data. All forces have been normalized based on body weight. It is apparent that the overall trends in forces of the cable corresponding to specific muscle before and after optimization closely resemble the subject's muscle force trends. Specifically, the cable force trends after optimization, particularly for the VAS muscle, align more closely with the subject's muscle activation level and timing compared to the initial cable force trends.

At the hip joint after optimization, the forces of cable simulating single-joint muscles ILPSO and GMAX exhibit slightly larger amplitude changes compared to the cable forces under the initial configuration. Conversely, the forces of cable corresponding to RF muscle displays smaller amplitude changes after optimization compared to the initial configuration. There is not much change in the amplitude of cable force corresponding to the HAMS muscle before and after optimization. After optimization, the amplitude change of cable force imitating the VAS muscle exatation is smaller than that under the original configuration, while the force of cable simulating the BFS muscle activation exhibits greater amplitude of change. The forces of cable corresponding to GAS and SOL muscles after optimization closely resemble the initial muscle forces in the first half, with significantly smaller amplitude of change afterward compared to the forces under the original configuration. The force of cable imitating TA muscle after optimization is greater than the force under the original configuration.

The cable forces of the elderly human model's left leg is presented in Figure 9B. The overall similarity in the trend of cable force changes before and after optimization in the left leg with respect to the subject is not as pronounced as in the right leg. Specifically, the trends in forces of cables corresponding to ILPSO, RF, HAMS, GAS, TA, and SOL muscles closely resemble the subject's muscle activation level and timing. However, for the GMAX, VAS, and BFS muscles, there is a noticeable phase difference in the cable force trends compared to the subject.

In terms of force changes, except for the cable corresponding to HAMS, BFS, and SOL muscles, the amplitudes of cable forces before and after optimization are smaller than those of the subject's muscle forces. Especially for ILPSO, RF, HAMS, GAS and SOL muscles, the trends and magnitudes of cable force changes after optimization are relatively close to muscles activation level and timing.

## 4 Discussion

While achieving a state in older adults that is similar to that of healthy adults individuals may not be realistic, some biomechanical data from individuals still holds certain reference value. In the design of human model, its height based on average elderly human data is shorter compared to the subject's height. Consequently, the forward speed, displacement, and the amplitude of the center of gravity's vertical oscillation are relatively smaller. In the planned motion, the left leg serves as the stance leg, while the right leg transitions from the stance leg to the swing leg with a larger range of motion. Therefore, the movement trends of the human model's left leg is closer to that of the subject. The joint angles of both ankles are directly mapped and remain unadjusted, hence the ankle joint angle trajectories are more akin to those of the subject.

In the Double Support Phase (DSP) of one walking gait, the Ground Reaction Forces (GRF) acts on both legs. In this paper, GRF is estimated based on the Zero Moment Point (ZMP) of subject, which is not entirely consistent with the human model, leading to not totally motion consistencies between the human model and the subject. However, when the human model's right leg leaves the ground, transitioning from DSP to Single Support Phase (SSP), the left leg remains in a stance position while the right leg swings. During this phase, GRF is entirely applied to the left leg. Consequently, the motion similarity between the human model and the subject is significantly higher.

Because the left leg remains the stance leg throughout and bears a greater load, the overall muscle force on the left leg tends to be larger. The calculation process for the subject's muscle forces in the right leg involves EMG signals from the muscles of the right leg, whereas the subject's muscle force in the left leg is entirely determined through biomechanical calculations. It is evident that the trends in cable force changes for the right leg aligns more closely with the human experiment data. Therefore, in subsequent work, increasing the number of surface electromyography (EMG) sensors can be considered to obtain more accurate muscle force data and better optimized cable forces.

When comparing the human model to the subject, it is observed that the human model has a slightly lower height but greater mass. When both entities move at similar speeds, the overall force experienced by the human model is slightly higher compared to that experienced by the subject. Notably, this difference is more pronounced in left leg. Furthermore, OpenSim modeling includes a rich set of muscles at the hip and knee joints, resulting in muscle force trends that do not entirely match. In contrast, at the ankle joint, where there are fewer muscles involved, the results tend to be more similar. It's worth noting that due to the optimization objective of minimizing energy consumption, cables corresponding to muscles like RF and HAMS, which cross both the hip and knee joints and have a large range of motion and force generation, experience significant reductions in forces after optimization. This also makes the output forces of the actuators be easily realized.

In future work, additional optimization objectives such as joint stiffness can be introduced into the optimization process for cable attachment points. Furthermore, more data from healthy elderly individuals will be considered to generate human-like motion trajectories. An analysis of the characteristics of muscle force calculations during human motion will be conducted, with the aim of enabling single-step calculations to provide more biomechanical support for the cable forces. Additionally, there are also plans to construct a lower limb exoskeleton prototype.

Despite some limitations in the method, the final results still indicate that the subject-specific method for determining the cable attachment points of a exoskeleton contributes to the generation of human-like motion with biomechanical and neuromuscular-control features.

## 5 Conclusion

This study aimed to investigate a personalized attachment point optimization method for cable-driven exoskeleton. The results indicate that, the elderly human model driven by the cable system exhibits cable forces similar to the muscle activation of healthy individual during similar movements. This similarity can facilitate muscle coordination and, ultimately, neurological recovery. Based on the analysis and results from the previous chapters, the following conclusions can be drawn.

The personalized attachment point optimization method successfully emulated the motion trajectories of experimental subjects, resulting in the exoskeleton device closely aligning with natural human coordination during motion. Estimation of joint torques in the exoskeleton also benefited from the application of personalized attachment points. Torque trends throughout motion closely matched those of experimental subject, particularly during transitions between double-leg support and single-leg support phases. Performance of the exoskeleton in terms of cable output forces and coordination improved through the optimization of personalized cable attachment points. Trends in the forces of cables corresponding to the key muscle groups closely aligned with those of experimental subject, especially in the optimized design where cable force changes better adhered to human motion neuromuscular-control features, such as muscle output forces and muscle activation timing sequence.

While this method still faces certain limitations, such as computational complexity and simplification of biomechanical models, these findings underscore the importance of personalized methods in the design of cable-driven exoskeletons. This approach holds promising prospects for applications in assisting older adults rehabilitation, providing valuable guidance for the cable-driven exoskeleton prototype realization.

## Data availability statement

The original contributions presented in the study are included in the article/Supplementary material, further inquiries can be directed to the corresponding authors.

## Ethics statement

The studies involving humans were approved by Institutional Ethics Review Board, Northwestern Polytechnical University.

## Author contributions

YC: Conceptualization, Data curation, Formal analysis, Methodology, Software, Visualization, Writing—original draft. WY: Conceptualization, Funding acquisition, Methodology, Project administration, Supervision, Writing—review & editing. AB: Conceptualization, Supervision, Writing—review & editing. DL: Data curation, Formal analysis, Investigation, Software, Writing—original draft. SK: Formal Analysis, Writing—original draft. RW: Conceptualization, Funding acquisition, Supervision, Writing—review & editing.
