# Supplementary material for: Towards Human-like Walking with Biomechanical and Neuromuscular Control Features: Personalized Attachment Point Optimization Method of Cable-Driven Exoskeleton
Source: Front Aging Neurosci. 2024 Feb 2;16:1327397. doi: 10.3389/fnagi.2024.1327397 (PMC10870425; doi:10.3389/fnagi.2024.1327397)
Supplement: Supplementary file 1 [file Data_Sheet_1.PDF]

Supplementary Material

1 SUPPLEMENTARY TABLES AND FIGURES

Muscle origin and insertion data are collected from eight males with an average height of 175 (±8.36) cm and an average age of 38.75 (±10.61) years, shown in Table S1, and the coordinate system diagram of muscle attachment points in the human body are shown in Figure S1. The distance between the hip joint and the knee joint averages 40.82 (±2.35) cm, while the distance between the knee joint and the ankle joint averages 38.00 (±2.37) cm.

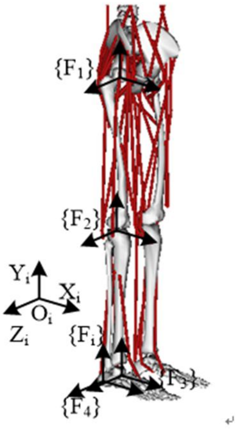

Figure S1. Coordinate system diagram of muscle attachment points in the human body.

Table S1. Subject muscle attachment points

Muscl	Origo(cm)	Ins(cm)
GM	[-8.16,5.40,-5.40]	[-0.73,29.30,4.69]
IL	[3.25,1.05,0.28]	[-1.93,33.17,1.16]
VL	[2.23,20.50,3.43]	[4.34,30.90,0.25]
BF	[-0.04,0.03,4.15]	[-0.33,31.68,4.99]
SO	[0.6,24.72,0.65]	[-4.06,-4.68,0.51]
TA	[1.82,20.91,0.68]	[6.82,-3.52,-1.47]
RF	[4.17,2.59,2.18]	[4.34,30.90,0.25]
HM	[-5.52,-5.44,-1.68]	[-0.54,26.83,-1.06]
GA	[-2.41,0.56,-2.04]	[-4.06,-4.68,0.51]

The initial attachment points and optimized attachment points are shown in the table S2.

Table S2. Initial attachment points and optimized attachment points

cable num	Initial origo(m)	Initial ins(m)	initial origo(m)	Optimized ins(m)
1	[0.1495 0.1325]	[0.0840 0.0893]	[0.1415 0.1210]	[0.1137 0.0800]
2	[0.0971 -0.1744]	[0.0541 -0.0780]	[0.0256 -0.0247]	[-0.1297 -0.0994]
3	[0.1341 0.1417]	[0.0749 0.1134]	[0.0939 0.0681]	[0.1092 0.0600]
4	[0.2104 -0.1538]	[0.0896 -0.0710]	[0.1589 -0.1418]	[-0.1693 -0.0748]
5	[0.2187 0.0890]	[0.0749 0.0934]	[0.2059 0.1162]	[0.1084 0.0547]
6	[0.4477 -0.0704]	[0.0667 -0.0533]	[0.4457 -0.0858]	[0.0897 -0.0403]
7	[0.4419 -0.0941]	[0.0637 -0.0906]	[0.4186 -0.0084]	[0.0677 -0.0375]
8	[0.1803 0.0682]	[0.0652 0.0182]	[0.1450 0.0355]	[0.0665 0.0833]
9	[0.1401 -0.0560]	[0.0637 -0.0906]	[0.1914 -0.0079]	[0.0801 -0.0551]
